# How to Target Spinal Metastasis in Experimental Research: An Overview of Currently Used Experimental Mouse Models and Future Prospects

**DOI:** 10.3390/ijms22115420

**Published:** 2021-05-21

**Authors:** Claudius Jelgersma, Peter Vajkoczy

**Affiliations:** Department of Neurosurgery, Charité—Universitätsmedizin Berlin, Corporate Member of Freie Universität Berlin and Humboldt-Universität zu Berlin, Charitéplatz 1, 10117 Berlin, Germany; peter.vajkoczy@charite.de

**Keywords:** spinal metastasis, in vivo, mouse, bone, multistep cascade, premetastatic niche

## Abstract

The spine is one of the organs that is most affected by metastasis in cancer patients. Since the control of primary tumor is continuously improving, treatment of metastases is becoming one of the major challenges to prevent cancer-related death. Due to the anatomical proximity to the spinal cord, local spread of metastasis can directly cause neurological deficits, severely limiting the patient’s quality of life. To investigate the underlying mechanisms and to develop new therapies, preclinical models are required which represent the complexity of the multistep cascade of metastasis. Current research of metastasis focuses on the formation of the premetastatic niche, tumor cell dormancy and the influence and regulating function of the immune system. To unveil whether these influence the organotropism to the spine, spinal models are irreplaceable. Mouse models are one of the most suitable models in oncologic research. Therefore, this review provides an overview of currently used mouse models of spinal metastasis. Furthermore, it discusses technical aspects clarifying to what extend these models can picture key steps of the metastatic process. Finally, it addresses proposals to develop better mouse models in the future and could serve as both basis and stimulus for researchers and clinicians working in this field.

## 1. Introduction

The spine is one of the most frequently affected systems in the context of metastatic processes [1]. Up to 70% of cancer patients develop secondary spinal metastasis, suffering from pain, pathological fractures and neurological deficits due to structural changes of the bone [1,2]. This topic becomes even more relevant given that anti-cancer treatments of primary tumors are improving and thus continuously leading to a better local tumor control. However, this increases the period in which spinal metastasis can occur [3]. As a consequence, the metastasis and not the primary tumor itself are becoming the leading cause of death [4,5]. Within the group of bone metastasis, the spine is the most affected [1]. The metastatic involvement of the spine was quantified in autopsy studies [6]. Metastases occurred in 90% from prostate cancer, 75% from breast cancer, 55% from melanoma, 45% from lung cancer and 30% from renal cancer [6]. Regarding the need for clinical treatment, the most frequently occurring spinal metastasis originate from lung (21%), prostate (19%) and breast (12%) cancer [7]. Metastatic spread is a highly complex process. It is based on Paget’s seed-and-soil theory which states that metastasis does not take place by chance and that tumor cells need a certain environment to be able to expand [8]. Over the years, various steps have been identified that are each equally important to develop metastasis. This process is known as the multistep cascade of metastasis [9,10,11]. These steps include events of how tumor cells become metastatic cells, spread to distant sites, and establish metastatic outgrowth. Key topics of the current metastatic research are the formation of a premetastatic niche in interaction with the primary tumor [12], tumor cell dormancy [13] as well as the influence of the immune system within the metastatic microenvironment [14]. Pari et al. concluded that recapitulating every aspect of the metastatic cascade in preclinical models is still one of the most challenging bottlenecks in the development of metastasis targeting therapies [15]. Therefore, it is of special interest to review the literature for current experimental models of spinal metastasis in order to decipher to what extent they mimic the metastatic cascade and whether they are integrating current key issues of metastatic research. This review focuses on mouse models, as mouse models are still one of the most powerful and indispensable tools in oncologic research [16]. This work is intended to provide an overview of the characteristics of these models and we will discuss distinctive mechanisms and aspects relevant to the process of spinal metastasis. Additionally, we will discuss prospects for the development of spinal metastasis models in the future.

## 2. Technical Aspects of Currently Used In Vivo Metastasis Mouse Models with Special Focus on Spine Metastasis

Literature research was performed with defined terms to look for spinal mouse models of entities that metastasize most frequently into spinal structures. These includes metastasis originating from prostate, breast, lung and kidney cancer as well as melanoma [6,7,17,18]. Search terms used in the pubmed database were “(spine OR spinal OR vertebral) AND (metastasis OR metastatic OR metast*) AND (mice OR mouse) AND (in vivo OR model) AND *respective entity*”. Studies were screened and considered if they had a clear focus on spinal metastases. These studies are included in Table 1 to provide an overview on the characteristics of currently used mouse models in the experimental research of spinal metastasis.

### 2.1. Inoculation Methods and Cell Lines

**Figure 1:**
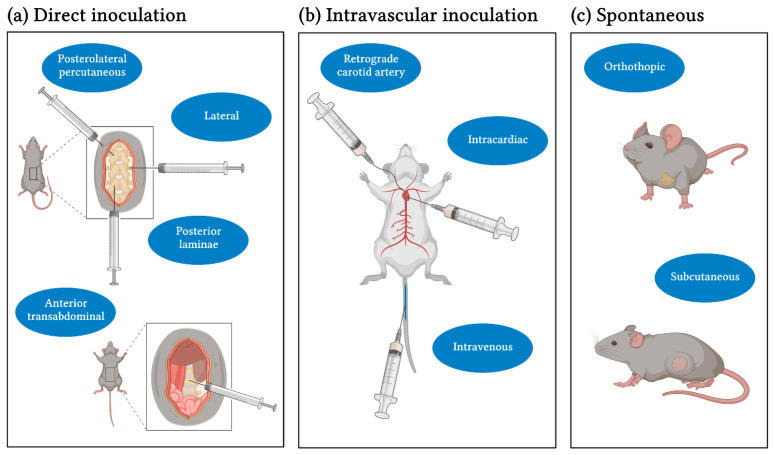
Overview of inoculation methods in experimental research of spinal metastasis. (**a**) Direct implantation methods lead to spinal metastasis with high reproducibility, but only the last steps of the metastatic cascade are depicted. Important advantages provided by direct inoculation methods are the rapid local growth which is limited to spinal structures. Therefore, it is well suited for the systematic investigation of clinical characteristics. (**b**) Using intravascular inoculation methods, earlier key steps including dissemination, circulation and extravasation mechanisms of tumor cells can be investigated. The preselection of the capillary bed through which the tumor cells pass first is to be mentioned as an advantage and disadvantage. This is realized by the choice of vessel (intravenously: lung → brain/body; intracardially: brain/body → lung; distal aortic arch: spine/body → lung → brain). (**c**) Spontaneous models are the only ones which are capable to recapitulate the whole metastatic process including early steps of the metastatic process. Therefore, they are suitable to investigate every step of the multistep cascade and current research questions as the formation of the premetastatic niche, organotropism to spine, tumor cell dormancy and the influence and regulating function of the immune system can be addressed. As they include primary tumor growth, they are the most realistic one and the influence of surgical resection on metastatic spread can be investigated. A disadvantage of these models, however, is the challenge to create reproducible metastases at a high frequency. Additionally, the investigation period can be limited due to metastasis to other sites which might occur prior to spinal metastasis (e.g., lung). Figure 1 was created with BioRender.com.

Various techniques have been developed over the years that are used to induce and model spinal and bone metastases, respectively. In order to gain insight into the technical aspects, special features of the individual inoculation methods are discussed in the following paragraph and summarized in Figure 1. Thus far, in the given overview of mouse models investigating spinal metastasis, intravascular injection techniques were used for cell inoculation primarily. These include intracardial [19,22,23,27,28,29,30,33], intraarterial [25,26,31,32] and intravenous [19,20,21] routes. Subcutaneous [38] or orthotopic injections at primary site [37] were used less frequently. Direct implantation methods include methods to implant tumor cell suspensions [34,36] or tumor pieces harvested from carrier animals [24,35] directly into vertebral structures.

The direct implantation was carried out in four different ways. Tumor cells were implanted through an anterior transabdominal approach into the vertebral body L3 [24], through a lateral approach into T10 [34], through a posterolateral percutaneous route into thoracolumbar junctions [36] or from posterior into the laminae and spinous process [35]. To carry out the anterior transabdominal inoculation, Tatsui et al. identified the left inferior kidney pole as an orientation mark after an abdominal midline incision in supine position [24]. After mobilization, the psoas muscle was dissected and retracted laterally to expose the L3 vertebral body [24]. A tumor piece was inserted after drilling a burr hole and prior to closing with a plug [24]. Apart from being technically challenging due to the abdominal incision and iliopsoas muscle dissection, this approach provides direct access to the vertebral body [24]. For the lateral injection, Tsung et al. placed all animals in decubitus position and carried out the skin incision in the extended line of the ulna and the olecranon to identify T10 [34]. Then, with a Hamilton syringe the tumor cell suspension was administered into the vertebral body from lateral, avoiding injury of the spinal cord and -canal [34]. Cossigny et al. immobilized the thoracolumbar junction between the fingers and forceps to inject the tumor cell suspension percutaneously posterolateral into the vertebral body [36]. For posterior implantation into the laminae and spinous process, Wang et al. accessed the bone, preparing muscle and fascia under the microscope to expose the targeted structure [35]. After hand drill decortication, the tumor tissue was inserted into the bone [35]. In comparison to the anterior transabdominal approach, direct inoculation methods from posterior, lateral or posterolateral are characterized by an easier handling. Thus, Wang et al. concluded a better accessibility from posterior while reducing the risk of neurological injury [35]. Cossigny et al. on the other hand, established the posterolateral approach, which was conducted percutaneously and therefore did not require microdissection [36]. It must be mentioned that, even if the handling was easy, in the end, a learning curve was needed, resulting in neurological injuries at the beginning [35,36].

Intravascular inoculation methods differ from direct implantation methods as they include the steps of the metastatic cascade related to the tumor cell homing via the blood stream as well as the extravasation at distant sites. For intravenous inoculation, the lateral tail vein was chosen, which is an easy and well establish procedure in animal research in general [19,20,21]. The intravenous route, however, leads to a significant loss of cells during the first pass through the lung. Thus, intracardial injection strategies have been developed. The principle of intracardiac injection is to mark orientation points (xiphoid and upper part of the sternum and the middle of both) and to puncture the left ventricle through the third intercostal space while penetration depth is controlled by the visible blood pulse in the syringe hub [39]. The third intravascular method was modified to circumvent not only lung, but also brain circulation by injecting cells into the distal aortic arch which was accessed by a retrogradely placed carotid artery catheter [25]. After exposing the left carotid artery and separating it from the vagal nerve, the artery was ligated and the catheter was placed, thus cells could directly disseminate into spinal structures [25].

Spontaneous metastasis models which focus on spinal metastasis are rare; only two studies have been published so far [37,38]. In fact, they resemble the clinical situation best and reflect the pre-conditioning of the spine microenvironment by soluble factors released from the primary tumor. In principle, tumor cells are implanted orthotopically or subcutaneously, and grow until they reach a size of 1 cm^3^. Then, the solid tumor is resected, and the animals are kept until they develop their metastasis spontaneously. For orthotopic implantation, the method depends on the targeted organ for primary tumor inoculation. To induce breast tumor metastases, Withana et al. injected tumor cells mixed with Matrigel into the mammary gland [37]. Another method to induce primary tumor growth before investigating metastasis is to inject tumor cells subcutaneously, for example into the rear thighs [38].

All models listed in this review are cell line-derived models, as the development of spontaneous tumors in small animals with corresponding metastasis is extremely rare and not suitable for metastasis research [40]. More than half of the listed studies in this review used xenograft models. In comparison with syngeneic models, the immune system is compromised to prevent graft versus host reaction when human cells are inoculated in mice [41]. This clearly represents a disadvantage of metastatic research, as the immune system plays a pivotal role in regulating and influencing metastasis [42]. The most common cell lines used in metastasis models focusing on spinal lesions are 4T1, TM40D, MDA-MB-231 cells in breast cancer, PC-3 in prostate cancer, A459, PC-9 and PC-14, NSCL murine line 1, LLC1 in lung cancer, ACHN in renal cancer and B16 and A2058 in melanoma (Table 1).

### 2.2. Time to Metastasis and Success Rate of Metastasis Establishment

The success rate of the establishment of spinal lesions is highly different between the listed studies (Table 1). Intravascular models achieved rates from 30 to 80%, whereas direct inoculation methods into vertebral structures showed a 100% success rate. The time until spinal metastasis developed, meaning detectable with imaging techniques or by clinical symptoms, was approximately 3–6 weeks [19,21,22,23,24,25,29,30,31,32,34,36,37,38] or longer [27,28,29,35] (Table 1).

### 2.3. Analysis Methods

There is a wide range of analysis methods that have been used to study metastatic processes and endpoints. One of the most challenging parts is to track tumor cells during the colonization process before they form a growing metastatic tumor. To investigate the development of metastasis longitudinally, Bioluminescence (BLI), Computer tomography (CT), Magnet Resonance Imaging (MRI) as well as Positron emission tomography (PET) have been used, with different advantages and disadvantages elsewhere described in detail [30,43]. To this day, the resolution of these imaging techniques is only at approximately 0.3–0.5 mm^3^ or worse [43]. Bioluminescence imaging is dependent on the successful preparation of cells before inoculation to express luciferase gene. Other methods are limited, because structural changes have to take place before metastases can be detected [30]. A promising method in this field is the use of PET-CT. In diagnostics of spinal metastasis in mice, Hu et al. demonstrated higher sensitivity, accuracy and negative predictive values for PET-CT imaging using 18F-FDG tracer in comparison to MRI because increased metabolic processes occurs before structural changes become detectable [30].

### 2.4. Consideration of Clinical Characteristics

Another important feature of experimental models of spinal metastasis is paying attention to the clinical assessment in particular the occurrence of limb paresis and plegia. This was most reproducible in models of direct tumor cell inoculation into vertebral structures [24,36]. Cossigny et al. mimicked evolving paraplegia caused by spinal tumors through direct percutaneous injection of tumor cells at the level of thoracolumbar junction [36]. Paresis and later paraplegia appeared 3–5 weeks after inoculation. Gait disturbances were first observed after 21–40 days of tumor growth. When unilateral hindlimb paralysis developed, time to paraplegia was only 24–72 h [36]. The clinical examination of the paraplegia was classified based on different scores. These scoring systems included first signs such as tail dragging, which is due to the decreasing muscle tone, or visible gait asymmetries. With progressing paralysis, hindlimb sweeping, unilateral paresis and finally complete paralysis could be observed in later stages [22,24,36]. Additionally, the severity of clinical symptoms correlated with the extent of spinal compression which was analyzed on histological sections [24]. Another method by which the arising neurological symptoms could be assessed more objectively was the application of catwalk examinations to investigate locomotion behavior [31]. This automated gait analysis allows determination of different parameters such as step count or walking speed [31,44].

### 2.5. Enhancing Metastatic Potential

In vivo selection is a well-known principle to enhance site specific metastasis by harvesting tumor cells from a specific site and re-implanting them into the next generation of animals after culturing [23,25]. This was demonstrated by Cai et al. with different tumor cell lines within three successive generations of in vivo selection. Interestingly, the group described multiple upregulated genes, which led to more aggressive and invasive subclones with a higher metastasis rate. These genes were related to migration, metastasis, adhesion and inflammation characteristics [23]. Another possibility is to genetically modify cells to express distinct receptors which have been shown to be involved in the spinal organotropism [27,45]. However, this also means that when target molecules have been defined, the natural process of homing and dissemination is being strongly interfered with. Beside these methods, there are more mechanistic approaches to increase the spinal metastasis frequency as well as the local specificity. One of the early discoveries was the relationship between the number of injected tumor cells and the number of metastases in bone in general [19]. The more cells inoculated, the more metastases could be induced [19]. Furthermore, the connection between the distribution of the metastases and the bone marrow content has been shown [19]. Another model used mechanical vena cava occlusion to increase spinal metastasis allowing tumor cells to preferentially spread via the venous route of Batson’s Plexus [20]. Vena cava was temporally occluded for 1 min during intravenous injection of tumor cells. Using this method, Harada et al. showed that significantly more tumor cells disseminated into vertebral bodies and the adjacent structures compared to injection without occlusion [20]. It is important to mention that this was found 5 min after injection. Interestingly, a subsequent study also showed differences in the long term establishment of metastasis [21]. Caval occlusion led to significantly more spinal lesions even 21 days after injection in the occlusion group, whereas no spinal lesion was found in the control group [21]. To inject tumor cells more specifically but still systemically at the same time, injection techniques which circumvent distinct organ systems by choosing the appropriate injection route are helpful. To bypass most of the lung perfusion, cells are injected into the left ventricle. To circumvent brain perfusion, retrograde carotid artery injection into the distal aortic arch was established [25]. With these methods, experiment-limiting lung metastasis as well as brain metastasis leading to falsified neurological symptoms could be reduced [25].

### 2.6. Mimicking of the Multistep Cascade of Metastasis

**Figure 2:**
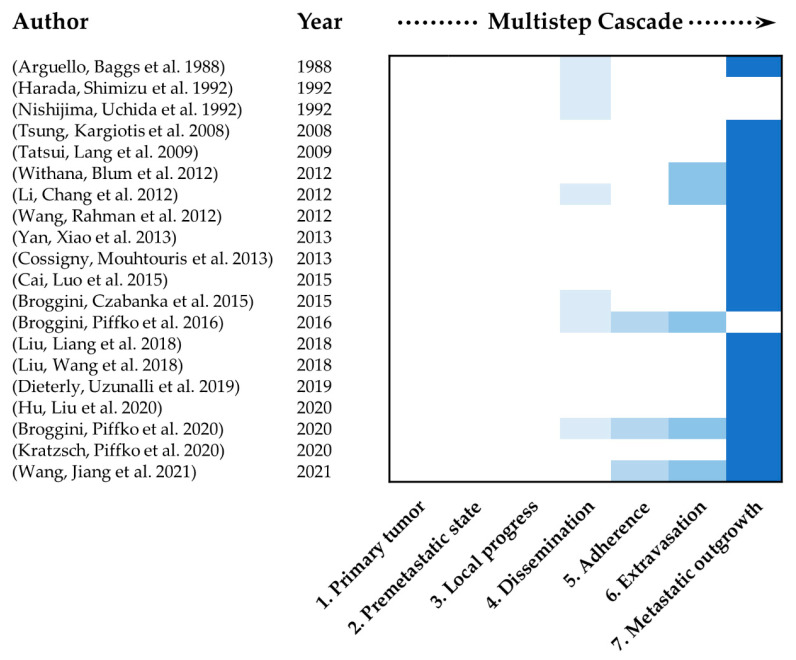
Allocation of the main study focus according to the corresponding step of multistep cascade of metastasis. Only studies listed in Table 1 of this review were included. Steps were defined as follows: 1. Primary tumor, 2. Premetastatic niche pre-colonization, 3. Local invasion and intravasation, 4. Circulation, dissemination and survival, 5. Adherence and arrest in a new organ, 6. Extravasation into the surrounding tissue, 7. Initiation and maintenance of growth and vascularization [9,10,11,46,47].

It is of particular interest to determine to what extent the different steps of the metastatic process are mimicked in the currently used mouse models. The metastatic process consists of multiple steps which are necessary to form metastasis. These multiple steps were defined previously and summarized as followed. 1. Selection of tumor cells with metastatic potential from the primary tumor, 2. conditioning of the premetastatic niche and pre-colonization, 3. local invasion and intravasation, 4. circulation and survival, 5. adherence and arrest in a new organ, 6. extravasation into the surrounding tissue and 7. initiation and maintenance of growth [9,10,11,46,47]. Figure 2 shows the main focus of the studies discussed in this review in regard to this multistep cascade of metastasis. None of the studies, included in this review, was able to map the entire metastatic process. Most of the studies only investigated late steps of the multistep cascade of metastasis. This mainly included dissemination of tumor cells and metastatic outgrowth. A key reason why these studies preferentially investigated later stages of the multistep cascade is that most models used intravascular application methods. This excludes all steps at the beginning of the cascade, in particular how cells carrying metastatic potential arise and migrate into the circulation as well as the interaction and preconditioning of the target organs and niches by the primary tumor. To be able to examine the first steps of metastasis, the formation of the primary tumor must be present. This can be achieved by using spontaneous orthotopic and subcutaneous inoculation models. Regarding the currently used models with special focus on spinal metastasis, only two model exist that use the subcutaneous or orthotopic inoculation method of tumor cells [37,38]. Thus, the focus of experimental spine metastasis research should be shifted to spontaneous pre-clinical models which are discussed in the future prospects section of this review.

## 3. Spine Organotropism

One of the most exciting questions is whether the process of metastasizing into the spine is spine specific, and which molecular mechanisms contribute to it. Only a few groups have focused on the metastatic ZIP code of the spine. One group from Shanghai identified CX3CL1 as a chemokine which promotes spine specific metastasis [27,33,45,48]. In human samples of spine metastasis, they found that upregulation of CX3CL1 is independent from the primary tumor [45]. In an experimental mouse model, the importance of this specific signaling pathway was shown [27]. Liu et al. found an enhanced frequency of spine metastasis in vivo when prostatic tumor cells overexpressed CX3CR1 [27]. Furthermore, spine specificity was increased with upregulated levels of CX3CL1 detected in healthy osseous tissue of the spine compared to healthy limbs [27]. The authors found Src/FAC signaling, activated by interaction with CX3CL1/CX3CR1, to be responsible for cell migration activities [27]. Additionally, the crucial role of CX3CL1 was shown within the process of trans-endothelial migration of cancer cells [48]. CX3CL1 regulates the vertebral micro-vascular barrier and induces disruption of vertebral marrow endothelial cells, thus promoting the extravasation of cancer cells with subsequent tumor growth specific to the spine [48]. Furthermore, ICAM-1 was induced by vertebral endothelial cells and then activated through CX3CL1, which facilitates the adhesion of circulating tumor cells and showed close interaction between these molecules [33]. As in vivo selection is described as a method to enhance site specific metastasis, Cai et al. investigated the gene expression signature associated with spine metastatic ability of in vivo selected cells [23]. The research group found that multiple genes were differently regulated in metastasis which are usually involved in many critical aspects of cancer development. Some of the respective genes play crucial roles in migration, metastasis, adhesion and inflammation thus contributing to optimal conditions for tumor growth [23]. Another explanation why metastasis occurs in the spine can be found in anatomical and physiological features. An investigation of the influence of dural tissue to the adjacent bone revealed that this microenvironment specifically supports tumor growth in close interaction with myeloid-derived suppressor cells (MDSC) which are known for their tumor promoting function [49]. Interestingly, the location of metastatic tumor growth within the vertebral body was mostly found in the posterior third close to the dura which might be the clinical correlation to the mechanisms mentioned before [49]. For this anatomical region, another study described that mesenchymal stem cells, which derived from the epidural adipose tissue promote metastatic outgrowth after activation by cancer cells [50]. These activated cells contribute to the preparation of a premetastatic niche, supporting metastatic tumor growth via the regulation of matrix metalloproteinases (MMP) and epithelial–mesenchymal transition (EMT) [50]. Taken together, this anatomical region with its corresponding influencing factors is largely spine-specific and could therefore be part of the explanation for the preference of circulating tumor cells to metastasize into the spine.

Other studies, in contrast, questioned the concept of spine organotropism. First, the idea that cells preferentially enter the spine via the so-called Batson venous plexus circumventing most of caval and portal circulation was disproved [51]. Originally Batson introduced this plexus as a fourth venous system which exists alongside with caval, pulmonary and portal venous system [51]. Veins of the plexus were characterized as valveless plexiform ramifications which infiltrates spinal bone structures and located in epidural space, within and extern of the vertebral bodies [51,52]. Located along the spine, these veins receive blood from cervical, thoracic, abdominal and pelvic regions [53]. Due to this characteristics and localization, intermittent reversal flow of blood can arise facilitating metastatic spread to spine [51,53]. However, Yuh et al. could not find different dissemination patterns among the venous (central Batson plexus) and the arterial (peripheral endplates) system within the vertebral body [54]. Additionally, dissemination studies with different types of tumor cells showed no difference of dissemination pattern between the spine and other bones [26]. This was proved by injecting microbeads, showing the same dissemination pattern into the spine and bone structures [26]. Additionally, tumor cells as well as microbeads were trapped by size restriction [26]. A recent study in humans observed that dissemination of tumor cells depends on red bone marrow content, supporting this finding [7]. The spinal bone with its bone marrow provides an optimal microenvironment with a high amount of growth factors and adhesion molecules [7,47]. In addition, due to the fenestrated sinusoidal organization of bone marrow endothelia, the intravasation and extravasation process is relatively straightforward [47].

If the metastatic process is taken as not spine-specific, but rather bone-specific, mechanisms of bone homing processes can be applied equally on spinal metastasis. There are several mechanisms which have already been identified to serve cancer cell homing to the bone [18,47,55]. Integrin αvβ3, stemness marker CD44 expressed by bone matrix and vascular cell adhesion molecule 1 (VCAM-1) which is constitutively expressed on bone marrow endothelial and stromal cells, lead to bone specific homing when respective molecules are expressed on tumor cells [47]. Another aspect is summarized by Ponzetti et al. who highlighted the similarity between the homing mechanisms of hematopoietic stem cells and cancer cells via signaling pathways like CXCR4. Therefore, CXCR4 is not only important for hematopoietic stem cell homing, but also for extravasation and interaction with bone marrow stromal components [18,47]. The important role of endothelial cells in homing mechanisms is mostly transduced by specific integrins or cell adhesion molecules (E-selectin ligand, β1 integrin, Rac1, CX3CL1/CX3CR1, ANXA2/ANXA2R) and is therefore crucial, as endothelial cells are the first cells in contact with cancer cells arriving in the bone marrow [18]. Nevertheless, other factors have been identified with distinct roles in promoting tumor progression after the establishment of metastatic colonization. CXCL17 for example is known to regulate different cancer promoting pathways such as angiogenesis and recruitment of specific immunocytes [56]. Liu et al. investigated the role in recruitment of M2 type macrophages which facilitate the formation of metastatic tumors in the spine. He discussed the importance of this pathway mainly for lung adenocarcinoma because they found higher levels of CXCL17 in this subtype compared to squamous cell carcinomas. This leads to the assumption that factors should be considered as specific to the primary tumor as well as the metastatic specific site. Fontanella et al. also concluded that intrinsic characteristics of tumor cells as well as the distant microenvironmental factors contribute to optimal metastatic growth [57]. In their review the research group described the predisposition of the bone for metastasis because of its high vascularization [57]. However, this alone is not the decisive factor. Rather this so called minimal residual disease, which could be found in 30% of breast cancer patients remaining in dormancy for several years, needs additional growth drivers to establish relevant tumor growth [57].

## 4. The Complexity of the Metastatic Microenvironment

It is undisputed that spinal bone structures or, bone in general, provide a tumor microenvironment that enables optimal conditions for the establishment and growth of metastases. Since most publications focus on investigations of the bone in general, this chapter also takes their findings into account. Systems that are involved in the formation of bone metastases are the bone structure itself with its different types of stromal cells and stem cell niches, the vascular system and the immune system [42,58]. Different molecular mechanisms have been identified to promote growth of metastasis in the bone microenvironment. Mostly, a distinction is made between osteolytic and osteoblastic pathological mechanisms [14,59]. However, a common mechanism is the disrupted balance between bone formation and bone loss in favor of tumor growth. The molecular mechanisms of metastasis are described elsewhere and are not the main objective of this article [58,59]. A special feature is the close relationship between the endosteal niche and endothelial niche [18,47]. Bone regeneration is closely related to interactions of osteoclastogenesis and angiogenesis, mediated by bone and endothelial cells [47]. These close interactions stand to reason, considering that bone marrow endothelial cells are known to promote dormancy in both hematopoietic stem cells as well as cancer cells and could additionally transdifferentiate into osteoblast-like cells leading to osteoblastic lesions [47]. The immune system contributes to optimal growth conditions by creating an immunosuppressive and privileged environment early on in the formation of an appropriate premetastatic niche [42]. Primary tumors release factors which include proinflammatory signals such as chemokines, cytokines, growth factors and extracellular vesicles to induce and regulate this process [60].

## 5. Future Prospects

Current issues in the research of the mechanisms of metastasis relate to the formation of the premetastatic niche, tumor cell dormancy and the involvement of immunological interactions [12,13,42]. Considering the complexity of the composition of the tumor microenvironment, it becomes clear that this question can only be answered in appropriate mouse models mimicking this cross-talk [15]. Thus, spontaneous and syngeneic mouse models are obligatory [61]. Half of the studies focusing on the spine (Table 1) had a xenogeneic background and used intravascular inoculation methods. This omitted all steps of the metastatic process that take place before tumor cells disseminate into the circulation. Consequently, the respective studies are only useful to investigate mechanisms and therapies for end stage metastatic disease. Spontaneous mouse models with orthotopic or subcutaneous primary tumors, from which the process of metastasis emerges, are required to study the mechanisms of the formation of the premetastatic niche. The Dissemination of metastatic cells starts early on, even during the development of preneoplastic lesions before the primary tumor becomes evident [62]. Disseminated cells could be detected in the spinal cord as early as seven days after inoculation of primary tumor cells in a spontaneous subcutaneous mouse model [38]. Additionally, mouse models should have a syngeneic background. A great advantage of syngeneic mouse models is the fully preserved functionality of the immune system, which is a basic requirement for investigating its influence and ability to regulate the metastatic process [61]. It has already been shown that immunological changes can be measured before metastasis can be detected in bones [61]. These changes mainly consisted of local bone marrow—as well as systemic immunosuppression [61].

However, the introduction of appropriate mouse models comes with major challenges as well. One finding of Takahashi et al. underpins the challenge of creating reproducible metastases at a high frequency in spontaneous metastatic models [63]. They showed that enhancement of bone metastasis by in vivo selection into the spine was possible using intravenous injection over three generations (increase of 63%) and that using these cells in a more spontaneous model via subcutaneous injection only led to an increased metastatic rate of 20% [63]. Focusing on techniques which lead to more specific metastasis to spinal bones might become a decisive factor in the future as metastases in other organs, especially of the lungs, often lead to an early termination of the experiment whilst shortening the possible investigation period [13,39]. Lung micro metastasis could already be detected on day 20 in a syngeneic orthotopic spontaneous mammary mouse model parallel to micro metastasis in bone [37]. Another issue that needs to be worked out is when and how primary tumors should be resected. On the one hand primary tumors can limit the life span of animals, resulting in a shortened investigation period. On the other hand, the resection itself can substantially influence the metastatic process [15,64]. For example, Buijs et al. showed that metastasis rate is correlated positively to the size of the primary tumor at the time of resection, using a syngeneic orthotopic mouse model of mammary cancer [64]. Furthermore, Zhang et al. pointed out that mechanisms of suppression by the primary tumor could lead to accelerated growth of metastases after resection when the inhibiting signal is no longer present [65]. Thus, alongside the establishment of spontaneous and syngeneic mouse models to investigate spinal metastasis, aspects of growth kinetics should be considered for future research to better understand these models.

It is also important to examine spinal metastases separately from bone metastases. Although both types of metastasis have a lot in common due to the bone structure, they can be influenced by their proximity to special structures, especially by the dura. In this context, Szerlip et al. showed the crucial role of dura as a biological active tissue which contributes to forming an appropriate niche and metastatic outgrowth by releasing factors to prepare an immunosuppressive environment by stimulation of bone marrow myeloid cells and promotes invasion and proliferation of metastatic cells [49].

Besides the need of investigating the involved mechanisms in early steps of metastatic process, it is also important to pay attention to treatments that are currently used in end stage metastatic disease but are not represented in preclinical spinal mouse models. This is mainly true for radiation therapy. Radiation therapy is already established in multidisciplinary treatment schedules, and advances have been made over the last years in optimizing techniques to achieve better outcomes [66,67]. Radiation modalities are different and include conventional external beam radiotherapy, stereotactic body radio therapy and stereotactic radiosurgery approaches [66,67,68]. Today, it represents a safe treatment option in a multidisciplinary context which allows effective and long-lasting local tumor control while its indications are expanding [67,68]. Thus, it would be of future interest, considering this option in preclinical models to gain more knowledge about mechanisms and optimal treatment schedules.

## 6. Conclusions

This review provides an overview of currently used mouse models of spinal metastasis and discusses to what extend the complexity of metastatic process is represented. Although spine metastasis is one of the most frequent sites of overall metastasis, specific models are rare. The discussion remains as to whether spinal metastasis is organ specific or whether metastasis occurs more frequently in the spine due to favorable conditions. The studies observed generally mimic only a few steps of the metastatic cascade in spinal metastasis and need to be improved. One reason is that mainly intravascular inoculation methods were used, which especially exclude the early steps of metastasis. Preclinical models must therefore be adapted and further developed in line with current research efforts relating to the formation of the premetastatic niche, the influence of the immune system and dormancy mechanisms of metastatic cells. Spontaneous syngeneic mouse models utilized to simulate bone metastasis are a good basis and can be transferred. Importantly, they include primary tumor formation as well as resection, therefore optimally mimicking clinical reality and should be established in preclinical mouse models of spine metastasis more frequently.

## Figures and Tables

**Table 1:** Characteristics of currently used mouse models in the experimental research of spinal metastasis.

Author, Year	Primary Origin	Cell Line	Model	Inoculation Method	Time to Metastasis and Success Rate	Analysis	Clinical Characteristics
**Intravascular Inoculation**							
Arguello, Baggs et al., 1988 [19]	Melanoma	G3.26 (B16)	Syngeneic, C57BL/6	IV, IC	14–20d, up to 100%	X-ray, Microscopic evaluation, Histology	No
Harada, Shimizu et al., 1992 [20]	Prostate	BrdU labelled PC-3	Xenograft, ICR athymic mice	IV ± caval occlusion	5 min, 75–100%	Histology	No
Nishijima, Uchida et al., 1992 [21]	Prostate	MBT-2	Syngeneic, C3H/He	IV	21d, 80%	Histology	Yes
Yan, Xiao et al., 2013 [22]	Breast	TM40D	Syngeneic, BALB/c	IC	35d, 60%	BLI, Digital X-ray, Histology, RT-PCR	Yes, #
Cai, Luo et al., 2015 [23]	Lung	PC-9, A549, NCI-H1299, NCI-H460, H2030	Xenograft, BALB/c nu/nu	IC	40d, success rates depending on cell line, A549L6: 80%	X-ray, Micro-CT, MRI, Histology	Yes, milestones [24]
Broggini, Czabanka et al., 2015 [25]	Melanoma	B16-luc and mB16-luc cells	Syngeneic, C57BL/6J	RCAI	19,5–29d	BLI, MRI, Histology	Yes
Broggini, Piffko et al., 2016 [26]	Lung, prostate, melanoma	B16-F1, LLC1, TRAMP-C2	Syngeneic, C57BL/6J	RCAI	-	Luciferase measurement	No
Liu, Liang et al., 2018 [27]	Prostate	PC-3	Xenograft, NOD/SCID mice	IC	42–56d, 45% (CX3CR1-overexpression)	PET-CT, Histology	No
Liu, Wang et al., 2018 [28]	Lung	A549 and SPCA-1	Xenograft, Balb/c nude mice	IC	42–56d, 30%	PET-CT, Histology	No
Dieterly, Uzunalli et al., 2019 [29]	Lung	A549-Br (Brain seeking variant)	Xenograft, Athymic Nude-Foxn1nu mice	IC	4–6 weeks, 39%	Histology	Yes
Hu, Liu et al., 2020 [30]	Lung	A549	Xenograft, Balb/c	IC	28–42d, 82,5% after 35d	MRI, PET-CT	No
Broggini, Piffko et al., 2020 [31]	Lung and melanoma	B16-F1 or LLC1 lung cancer cells	Syngeneic, Tamoxifen-inducible endothelial-specific ephrin-B2 knockout mice (efnb2iΔEC) and efnb2lox/lox	RCAI	15d	BLI, MRI, Intravital fluorescens video microscopy, Histology	Yes, catwalk experiments
Kratzsch, Piffko et al., 2020 [32]	Melanoma	B16-F1	Syngeneic, C57BL/6J mice	RCAI	14–21d, 78%	BLI, MRI, Histology	Yes
Wang, Jiang et al., 2021 [33]	Lung	A549	Xenograft, NOD/SCID mice	IC	Approx. 14d	BLI, CT, Histology, PCR	No
**Direct Inoculation**							
Tsung, Kargiotis et al., 2008 [34]	Melanoma	Human melanoma cell line A2058	Xenograft, Nude mice	Laterally into T10	18d, 100%	Histology	Yes
Wang, Rahman et al., 2012 [35]	Kidney	Human renal carcinoma cell line, ACHN	Xenograft, NOD/SCID	Spinous process and lamina	After 12 weeks, 33% paraplegia	Histology	Yes
Tatsui, Lang et al., 2009 [24]	Lung	PC-14	Xenograft, Athymic Nude mice	Anterior transabdominal injection into L3	28d	Histology	Yes, ##
Cossigny, Mouhtouris et al., 2013 [36]	Breast and prostate	PC-3MDA-MB-231	Xenograft, BALB/Nu-Nu athymic mice	Percutaneously upper lumbar spine	21–35d, 100%	X-ray, Micro-CT Histology	Yes, ###
**Spontaneous Mouse Models**							
Withana, Blum et al., 2012 [37]	Breast	4T1.2	Syngeneic, BALB/c	Orthotopic	30d	Fluorescens Tomography, Histology, qRT-PCR	No
Li, Chang et al., 2012 [38]	Lung	NSCL murine Line 1	Syngeneic, BALB/cByJ mice	SC	7d, micrometastasis	BLI, Histology	No

IV, intravenous; IC, intracardiac; SC, subcutaneous; RCAI, retrograde carotid artery injection; #, three categories: normal: animal could walk; partial paralysis: animal could stand but walk; complete paralysis: animals have little or no limb activity [22]; ##, milestones: 1. tail dragging 2. dorsal stepping, 3. hindlimb sweeping 4. Paraplegia [24]; ###, Grade 0 = normal, Grade 1 = gait disturbance, Grade 2 = complete unilateral hindlimb paralysis, Grade 3 = complete bilateral hindlimb paralysis with mobilization only possible using the forelimbs [36].

## Data Availability

Not applicable.
